# Quercetin Lowers Plasma Triglycerides Accompanied by White Adipose Tissue Browning in Diet-Induced Obese Mice

**DOI:** 10.3390/ijms19061786

**Published:** 2018-06-16

**Authors:** Eline N. Kuipers, Andrea D. van Dam, Ntsiki M. Held, Isabel M. Mol, Riekelt H. Houtkooper, Patrick C.N. Rensen, Mariëtte R. Boon

**Affiliations:** 1 Department of Medicine, Division of Endocrinology, Leiden University Medical Center, Post Zone C7Q, P.O. Box 9600, 2300 RC Leiden, The Netherlands; e.n.kuipers@lumc.nl (E.N.K.); andrea.vandam@ocdem.ox.ac.uk (A.D.v.D.); i.m.mol@lumc.nl (I.M.M.); p.c.n.rensen@lumc.nl (P.C.N.R.); 2 Einthoven Laboratory for Experimental Vascular Medicine, Leiden University Medical Center, P.O. Box 9600, 2300 RC Leiden, The Netherlands; 3 Laboratory Genetic Metabolic Diseases, Amsterdam Gastroenterology and Metabolism (AG&M) institute, Academic Medical Center, 1105 AZ Amsterdam, The Netherlands; n.m.held@amc.uva.nl (N.M.H.); r.h.houtkooper@amc.uva.nl (R.H.H.)

**Keywords:** brown adipose tissue, browning, obesity, quercetin, triglycerides, white adipose tissue

## Abstract

Obesity and dyslipidemia are major risk factors for the development of cardiovascular diseases (CVD). Quercetin, a natural flavonoid, lowers plasma triglycerides (TG) in human intervention studies, and its intake is associated with lower CVD risk. The aim of this study was to elucidate the mechanism by which quercetin lowers plasma TG levels in diet-induced obesity. C57Bl/6J mice received a high-fat diet (45% of calories derived from fat) with or without quercetin (0.1% *w*/*w*) for 12 weeks. Quercetin decreased plasma TG levels from nine weeks onwards (−19%, *p* < 0.05), without affecting food intake, body composition, or energy expenditure. Mechanistically, quercetin did not reduce intestinal fatty acid (FA) absorption. Rather, quercetin induced a slight reduction in liver *Apob* expression (−13%, *p* < 0.05), which suggests decreased very-low density lipoprotein-TG production. Interestingly, quercetin also markedly increased the uptake of [^3^H]oleate, which was derived from glycerol tri[^3^H]oleate-labeled lipoprotein-like particles by subcutaneous white adipose tissue (sWAT, +60%, *p* < 0.05). Furthermore, quercetin also markedly increased mRNA expression of *Ucp1* (+229%, *p* < 0.05) and *Elovl3* (+138%, *p* < 0.05), specifically in sWAT. Accordingly, only quercetin-treated animals showed uncoupling protein-1 protein-positive cells in sWAT, which is fully compatible with increased browning. Taken together, the TG-lowering effect of quercetin may, at least in part, be due to increased TG-derived FA uptake by sWAT as a consequence of browning.

## 1. Introduction

Cardiovascular diseases (CVD) are the number one cause of death worldwide. Obesity and dyslipidemia are major risk factors for developing CVD [1,2,3]. Super foods and other natural products, including flavonoids, have become increasingly popular as potential strategies to target these risk factors. Quercetin is a naturally occurring flavonoid that is present in fruits and vegetables [4]. Interestingly, epidemiological studies show that increased quercetin intake is associated with a reduced risk for developing CVD [5]. In a preclinical study, quercetin supplementation indeed reduced atherosclerosis development [6]. Furthermore, quercetin supplementation attenuates body weight gain [7,8,9] and lipid deposition in the liver [10,11], as well as white adipose tissue depots [12] of animals on a high-fat diet. In addition, quercetin lowers plasma triglyceride (TG) levels in mice [7,9,13] and humans [14], thereby targeting another risk factor for CVD.

The exact mechanism underlying the TG-lowering effect of quercetin and the organs involved have not yet been elucidated. Interestingly, a recent in vitro study showed that quercetin induces the browning of 3T3-L1 white adipocytes [15], as they adapted a more brown-like phenotype, including higher mRNA expression of the uncoupling protein 1 (UCP-1) [16]. Of note, we recently showed that browned white adipose tissue (WAT) depots increase their uptake of TG-derived fatty acids (FA) from the circulation compared with the same WAT depots in mice that lack the browning stimulus [17,18]. In addition, brown adipose tissue (BAT) is a major contributor to TG metabolism [19,20]. When activated by e.g., cold, BAT enhances the oxidation of intracellular FA in both mice [17] and humans [21], which causes the rapid depletion of intracellular TG stores. As a consequence, BAT takes up large amounts of TG-derived FA in a lipoprotein lipase (LPL)-dependent manner and combusts these to generate heat in a process that is dependent on mitochondrial UCP-1 [20,22].

Whether BAT and (browned) WAT are involved in the beneficial effects of quercetin with respect to the reduction of plasma TG remains to be determined. Therefore, in the current study, we aimed at elucidating the mechanism by which quercetin lowers plasma TG levels by studying whole body lipid metabolism with the focus on BAT and WAT in diet-induced obese mice.

## 2. Results

### 2.1. Quercetin Reduces Plasma Triglyceride Levels without Affecting Body Composition, Food Intake, and Energy Expenditure

**Figure 1:**
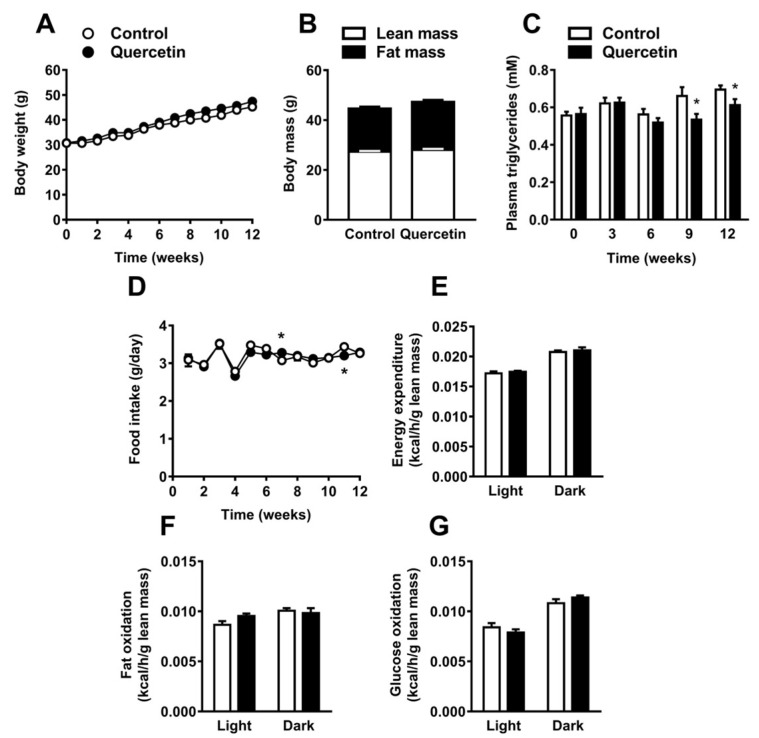
Quercetin reduces plasma triglyceride levels without affecting body composition, food intake and energy expenditure. C57Bl/6J mice were fed a high-fat diet (HFD) +/− quercetin (0.1% *w*/*w*) for 12 weeks. Body weight was measured weekly (**A**). Body composition was determined (**B**). Plasma triglycerides were determined before and every three weeks during the intervention (**C**). Food intake was determined weekly (**D**). In the third week of treatment, animals were placed in fully automatic metabolic cages for an assessment of total energy expenditure (**E**), fat oxidation (**F**), and glucose oxidation (**G**). Data are represented as mean ± SEM (*n* = 8–10), * *p* < 0.05 versus control.

In order to assess the effect of quercetin on body composition, plasma lipids, and energy balance, C57Bl/6J mice were fed a high-fat diet (HFD) with or without 0.1% (*w*/*w*) quercetin for 12 weeks. Quercetin neither affected body weight (mean body weight after 12 weeks 45.2 ± 2.5 g in control and 47.4 ± 3.3 g in quercetin-treated animals; Figure 1A) nor body composition with respect to lean and fat mass (Figure 1B). In line with these data, the organ weight of spleen, gonadal WAT (gWAT), and interscapular BAT (iBAT) at necropsy were unaffected by quercetin. We only found liver weight to be increased (+13%, *p* < 0.05; Figure S1A). Of note, quercetin lowered plasma TG levels from nine weeks onwards (up to −19%, *p* < 0.05; Figure 1C). Furthermore, quercetin treatment increased plasma total cholesterol levels—mainly due to increased cholesterol in the high density lipoprotein fraction (Figure S1B,C)—decreased plasma free FA (FFA) levels, and did not affect plasma glucose (Figure S1D,E). The lowering in plasma TG levels by quercetin could not be explained by a lower daily food intake, since daily food intake was not persistently affected by quercetin (Figure 1D). To determine whether quercetin influenced whole body energy expenditure and substrate utilization, mice were placed in metabolic cages in the third week of treatment. Quercetin did not affect total energy expenditure (EE), fat, or glucose oxidation (Figure 1E–G). The respiratory exchange ratio (RER) and physical activity were also unaltered (Figure S1F,G). Taken together, quercetin reduced plasma TG without affecting body weight, body composition, food intake, and energy expenditure. 

### 2.2. Quercetin Reduces Hepatic ApoB Expression and Increases Uptake of TG-Derived FA by sWAT

**Figure 2:**
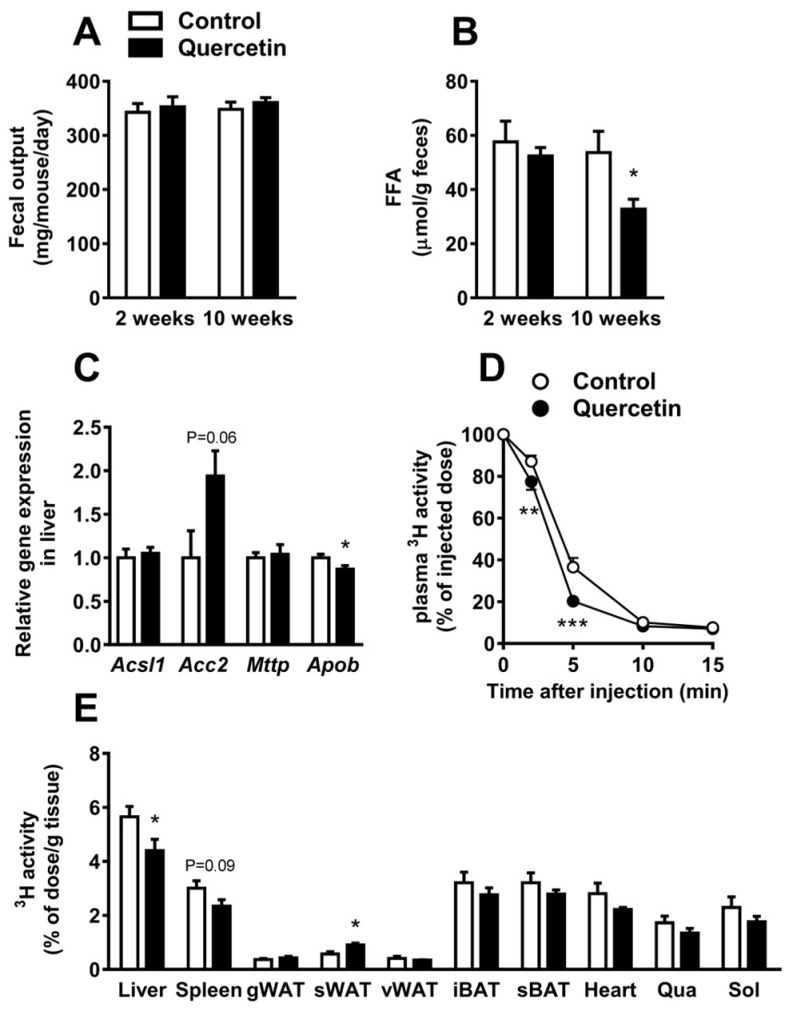
Quercetin reduces hepatic apolipoprotein B (*Apob)* expression and increases the uptake of triglycerides (TG)-derived fatty acid (FA) by subcutaneous white adipose tissue. In week 2 and week 10 of the intervention, 24 h feces was collected (**A**) and used to determine fecal free fatty acid (FFA) concentration (**B**). Gene expression in the liver was determined by qRT-PCR for acyl-CoA synthetase long-chain family member 1 (*Acsl1)*, acetyl-CoA carboxylase 2 (*Acc2*), microsomal triglyceride transfer protein (*Mttp*), and *Apob* (**C**). After 12 weeks, mice were injected with glycerol tri[^3^H]oleate-labeled lipoprotein-like particles, and clearance from plasma (**D**) and uptake per gram organ (**E**) were determined by ^3^H-activity analysis. Data are represented as mean ± SEM (*n* = 8–10); the expression of genes was corrected for the reference gene *β2-microglobulin*, * *p* < 0.05, ** *p* < 0.01, *** *p* < 0.001 versus control. (g,s,v)WAT: gonadal, subcutaneous, visceral white adipose tissue; (i,s)BAT: interscapular, subscapular brown adipose tissue; qua: quadriceps muscle; sol: soleus muscle.

Plasma TG levels are determined by the balance between intestinal TG uptake, hepatic very-low density lipoprotein (VLDL)-TG production, and VLDL-TG clearance by LPL-expressing peripheral organs. We first assessed total fecal output, measured over 24 h, which was unaltered after two and 10 weeks of quercetin treatment (Figure 2A). Interestingly, FFA content in feces was reduced after 10 weeks of quercetin treatment (−39%, *p* < 0.05; Figure 2B), suggesting enhanced rather than decreased intestinal TG absorption. We next focused on the effect of quercetin on liver lipid metabolism. Although quercetin increased liver weight (Figure S1A), quercetin did not affect liver lipid content (Figure S2A,B). The activation of xenobiotic sensing nuclear receptor pregnane X receptor (PXR), as the underlying cause for the increased liver weight, was excluded, since *Pxr* expression and the expression of the PXR target gene *Cyp3a11* remained unchanged (Figure S2C). The hepatic expression of lipid synthesis and oxidation marker acyl-CoA synthetase long-chain family member 1 (*Acsl1*) was unaffected, and lipid synthesis marker acetyl-CoA carboxylase 2 (*Acc2*) tended to be increased after quercetin treatment (Figure 2C).

To assess the effect of quercetin on VLDL-TG production, we determined the mRNA expression of hepatic microsomal triglyceride transfer protein (*Mttp)* and apolipoprotein B (*Apob*), which are both involved in hepatic VLDL assembly and secretion (Figure 2C). Of note, quercetin decreased *Apob* expression (−13%, *p* < 0.05; Figure 2C), which may indicate lower VLDL production.

To investigate whether quercetin reduced plasma TG levels due to an increased clearance of TG-rich lipoproteins, we determined the kinetics of intravenous injected [^3^H]triolein (TO) labeled TG-rich lipoprotein-like particles, and studied the plasma clearance of [^3^H]TO and organ uptake of [^3^H]TO-derived [^3^H]oleate in mice treated with quercetin for 12 weeks. The plasma clearance of [^3^H]TO was slightly accelerated in the quercetin-treated group (Figure 2D). An uptake of ^3^H per gram organ was decreased in the liver (−22%, *p* < 0.05), but this effect disappeared when data were corrected for whole organ weight (Figure S2D). Interestingly, quercetin markedly increased (+60%, *p* < 0.05; Figure 2E) the uptake of ^3^H per gram organ by subcutaneous WAT (sWAT), while the uptake by the two studied classical BAT depots (interscapular and subscapular) were not changed. All in all, our data suggest that quercetin decreases plasma TG levels by decreasing VLDL-TG production and increasing TG-derived FA uptake by sWAT. 

### 2.3. Quercetin Increases Ucp1 Gene Expression Specifically in sWAT

**Figure 3:**
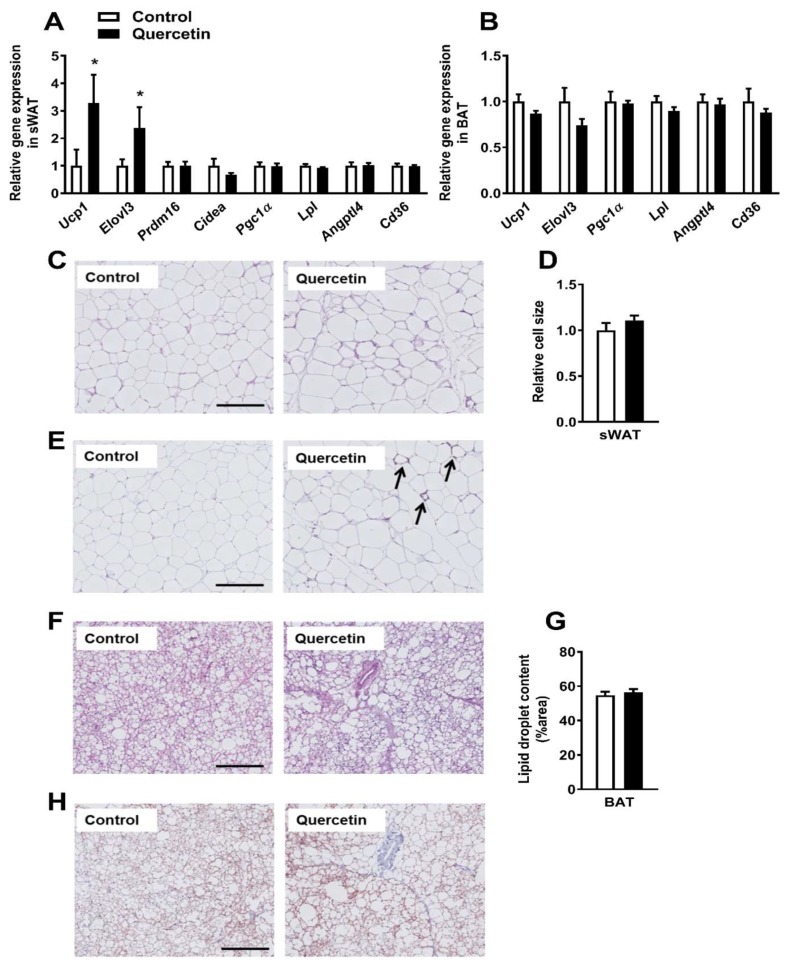
Quercetin increases uncoupling protein-1 (*Ucp1)* gene expression specifically in subcutaneous white adipose tissue. Gene expression in sWAT (**A**) and BAT (**B**) was determined by qRT-PCR. Hematoxylin and eosin (H&E) staining was performed on paraffin-embedded sWAT sections, and representative pictures are shown (**C**). Pictures were analyzed in ImageJ to determine the relative cell size (**D**). sWAT sections were stained for UCP-1 (arrows indicate UCP-1 positive cells, **E**) as well. BAT sections were stained for H&E (**F**) and used to quantify lipid droplet content in ImageJ (**G**). BAT sections were also stained for UCP-1 (**H**). Data are represented as mean ± SEM (*n* = 8–10); the expression of genes was corrected for the reference gene β*2-microglobulin* (sWAT), *Gapdh*, and *Hprt* (BAT), * *p* < 0.05 versus control. Bars (**C**,**E**,**F**,**H**) indicate 200 µm.

Since the increased uptake of TG-derived FA by sWAT might point to the increased browning of this depot [17,18], we next determined whether quercetin had induced browning of sWAT. Indeed, quercetin markedly increased the expression of *Ucp1* in sWAT (+229%, *p* < 0.05) as well as the brown fat marker fatty acid elongase 3 (*Elovl3*, +138%, *p* < 0.05, Figure 3A). The expression of other markers of BAT-like metabolism, including the transcriptional coregulators PR domain containing 16 (*Prdm16*) and cell death-inducing DFFA-like effector A (*Cidea*), the central inducer of mitochondrial biogenesis, peroxisome proliferator-activated receptor γ coactivator 1 (*Pgc1α*), and markers involved in lipid uptake, including lipoprotein lipase (*Lpl*), the LPL regulator angiopoietin-like 4 (*Angptl4*), and fatty acid translocase (*Cd36*) were unaltered in sWAT (Figure 3A). Also, the p-AMP-activated protein kinase (AMPK) (Thr172)/AMPK ratio in sWAT did not differ between quercetin and the control group (Figure S3A). Furthermore, quercetin did not increase the expression of *Ucp1*, *Elovl3*, *Pgc1α*, *Lpl*, *Angptl4,* and *Cd36* in BAT (Figure 3B), gWAT (Figure S4A), and visceral WAT (vWAT, Figure S4B). To verify browning on a morphological level, sWAT sections were stained with hematoxylin and eosin (H&E) (Figure 3C), and examined for UCP-1 (Figure 3E). While relative cell size did not differ between the two treatment groups (Figure 3D), quercetin did induce the presence of UCP-1 positive cells (indicated by the arrows), while these were completely absent in the control animals (Figure 3E). We also attempted to measure UCP-1 content in sWAT via western blot, but the content appeared too low to draw conclusions (data not shown). Quercetin did not increase mitochondrial abundance, since only the mitochondrial DNA versus nuclear DNA (mtDNA/nDNA) ratio of *16s/Hk2* tended to be increased (*p* = 0.08, Figure S3B) in the quercetin-treated animals, while ratios of *Cox2/Ucp2*, *Cox2/Hk2,* and *16S/Hk2* were unaffected. In line with the qRT-PCR data, quercetin did not affect the histological appearance and lipid droplet content of BAT (Figure 3F,G), gWAT (Figure S4C,D), and vWAT (Figure S4E,F). Also, quercetin did not affect the UCP-1 content in BAT (Figure 3H) or UCP-1 protein content as measured via western blot (data not shown). All in all, our data shows that quercetin increases the expression of BAT markers and UCP-1 content specifically in sWAT.

## 3. Discussion

**Figure 4:**
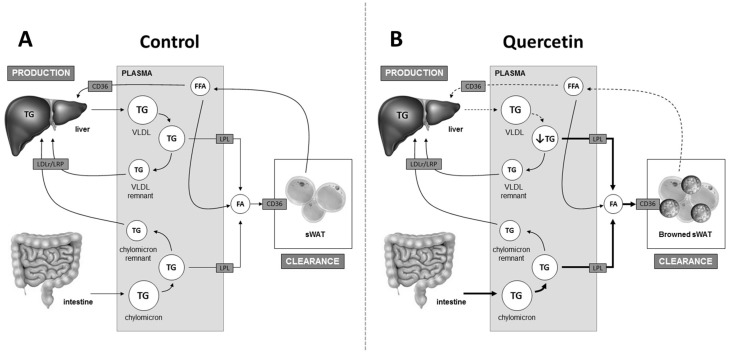
Proposed mechanism by which quercetin lowers plasma triglycerides (TG). Plasma TG levels are regulated by intestinal TG absorption, hepatic very-low density lipoprotein (VLDL)-TG production, and the clearance of TG-derived fatty acids (FA) by peripheral organs such as sWAT in a process that is dependent on lipoprotein lipase (LPL) (**A**). Quercetin induces the browning of sWAT, resulting in an increased uptake of TG-derived FA by sWAT. This may contribute to the lowering of plasma TG levels. Moreover, quercetin may also lower lipolysis from sWAT, which may contribute to lower plasma free FA (FFA) levels and lower VLDL-TG production by the liver, also contributing to lower plasma TG levels. Lastly, quercetin increases the uptake of lipids by the intestine, which apparently does not counteract the reduction in plasma TG levels (**B**). Bold arrows indicate increased flux; dashed arrows indicate decreased flux. LDLr: low-density lipoprotein receptor; LRP: low-density lipoprotein receptor-related protein.

Previous studies in humans and animals have shown that quercetin is a possible new agent to improve metabolic health, since it lowers plasma TG levels [7,13,14]. The organs involved in the TG-lowering effect of quercetin remained obscure so far. In the current study, we demonstrated that quercetin reduces plasma TG as well as FA levels, which is accompanied by an increased flux of TG-derived FA towards sWAT, but not BAT, and the induction of browning of this depot. Quercetin probably lowers VLDL-TG production, as indicated by lower *Apob* expression. We conclude that the TG-lowering effect of quercetin may, at least in part, be due to increased TG-derived FA uptake by sWAT as a consequence of browning (Figure 4). 

Our finding that quercetin lowers plasma TG levels has been repeatedly shown by others [7,11,13,23]. Of note, in all of these studies, quercetin lowers plasma TG levels, even though different concentrations (ranging from 0.025% to 0.33% *w*/*w*) of quercetin were added to a high-fat and/or high-fructose diet. This indicates that quercetin is a potent modulator of plasma TG levels, and exerts metabolically beneficial effects at a wide dose range. In contrast to our study, several of these studies also reported a reduction in body weight following quercetin treatment, again in a dose range [7,11]. This discrepancy might be explained by a difference in the age of the animals at the start of the intervention. Studies that describe weight reduction after quercetin treatment used animals of 4–5 weeks of age, which are not yet developed into adult mice, and might therefore be more susceptible to the body weight changes caused by the intervention compared to the nine-week-old animals that were used in this study. 

To gain insight into the mechanism underlying the TG-lowering effect of quercetin, we determined the effect of quercetin on several metabolic organs and performed kinetic studies to assess FA fluxes. To assess whether quercetin affected intestinal FA absorption, we measured fecal FA output. A previous study in Caco-2 cells, which mimic human intestinal epithelial cells, shows that quercetin increases ^3^H-FA uptake, but only when initial ^3^H-FA uptake was reduced under conditions of oxidative stress [24]. However, fecal FA in our study was lower rather than higher in the quercetin-treated group. This suggests higher rather than lower intestinal FA absorption, which therefore cannot explain the observed reduction in plasma TG levels. Interestingly, we found that quercetin reduced liver *Apob* expression, which might indicate lower VLDL production. In support of this hypothesis, Gnoni et al. [25] found that the stimulation of isolated rat hepatocytes with quercetin decreases TG synthesis and VLDL-TG formation due to lower ACC and diacylglycerol acyltransferase activity. A lower VLDL-TG production might underlie the increase in liver weight in the quercetin-treated animals by inducing a trend toward higher TG accumulation. Of note, this is in contrast to previous literature, in which liver weight was shown to be unaffected [26] or even decreased, due to a reduction in liver TG [11]. Next to a direct effect of quercetin on hepatocytes, a lower VLDL production in vivo might also be the consequence of lower FA flux toward the liver due to reduced FA liberation by lipolysis in WAT. In line with this, we showed that quercetin lowered plasma FFA levels, although we did not find any effect of quercetin on the expression of markers of lipolysis in white adipose tissue (not shown). Rather, others have shown that quercetin increases lipolysis in white adipocytes [15,27]. Alternatively, lower plasma FFA levels might also be a consequence of increased FFA uptake by adipose depots, since not only TG-derived FA, but also FFA, can be taken up by BAT [28] and possibly also by browned white adipose tissue.

Our kinetic study using labeled lipoprotein-like TG-rich particles showed that quercetin increases the uptake of TG-derived FA specifically by sWAT, which is the depot that is especially prone to browning [29]. This metabolic effect, along with increased *Ucp1* and *Elovl3* expression and the presence of UCP-1 positive cells in sWAT, is fully compatible with increased browning. In agreement with our results, Lee et al. [15] recently showed that onion peel, with quercetin as a main component, induces *Ucp1* expression in the white adipose tissue of mice. However, effects on plasma TG levels were not reported in that study. Of note, we found that quercetin solely increased *Ucp1* expression and TG-derived FA uptake in sWAT, and did not affect uptake by BAT in our study. Adipose depot-specific responses to intervention have been observed before. For instance, mice with genetically increased levels of bone morphogenetic protein 4 are protected from obesity due to a phenotype with sWAT browning, despite BAT accumulating lipids and adapting a beige/brite phenotype [30]. It can also be questioned whether adipose tissue browning has the capacity to reduce plasma TG levels, since the UCP-1 content in sWAT is quite low. Interestingly, inactivating the classical BAT depot by the selective inhibition of lipid droplet lipolysis induces WAT browning and lowers TG levels in peripheral organs, which indeed demonstrates the potency of browning per se to affect TG distribution [31], and possibly also to reduce plasma TG levels. Our data thus suggest that quercetin induces browning that could, at least in part, be responsible for the improved plasma TG levels (Figure 4). Future studies are evidently needed to clarify the extent to which adipose tissue browning contributes to the metabolically beneficial effects induced by quercetin. 

How quercetin might induce the browning of sWAT has not yet been fully elucidated. Lee et al. [15] proposed a mechanism by which quercetin acts directly on the adipocyte, and induces browning at least in part via the AMP-activated protein kinase (AMPK)/sirtuin1/PGC1α pathway. Activation of this pathway results in increased *Pgc1α* expression, suggesting an increase in mitochondrial biogenesis. Although quercetin tended to slightly increase one of the four mtDNA/nDNA ratios in sWAT in our study, quercetin did not affect the sWAT p-AMPK/AMPK ratio and *Pgc1α* expression. Interestingly, quercetin is known as a phytoestrogen because of its binding capacity to the estrogen receptor (ER) [32]. Pharmacological activation of the ER subtype β (ER-β) in mice reduces body weight and adiposity with concomitant enhanced oxygen consumption and elevated core body temperature [33]. Moreover, ER-β ligand LY3201 induces browning of sWAT by modulating the sympathetic ganglia and the adipocytes directly [34]. The mechanism underlying the quercetin-induced browning of specifically sWAT, and the possible involvement of the ER-β remains to be determined, and would be an interesting avenue to pursue in future studies. In addition, whether quercetin induces browning in humans, and whether this mediates the improvement of TG metabolism, remains to be studied. 

In conclusion, our study shows that quercetin induces browning of sWAT, which results in an increased flux of TG-derived FA towards sWAT and which may, at least in part, underlie the TG-lowering effect of quercetin (Figure 4). The mechanism behind these effects and the ability of quercetin to induce browning in humans remains to be determined.

## 4. Materials and Methods

### 4.1. Animals and Diet

Male C57Bl/6J mice (*n* = 18, Charles River Laboratories, Wilmington, MA, USA), nine weeks of age, were placed on a run-in high-fat diet (HFD, 45% of calories derived from fat, D12451, Research Diets, New Brunswick, NJ, USA) for three weeks. Next, mice were randomized based on body weight and plasma TG levels to receive a HFD with or without quercetin (0.1% *w*/*w*) for 12 weeks. Mice were housed individually under standard conditions with ad libitum access to food and water. Mouse experiments were performed in accordance with the Institute for Laboratory Animal Research Guide for the Care and Use of Laboratory Animals and have received approval from the University Ethical Review Board (DEC14089, Leiden University Medical Center, Leiden, The Netherlands).

### 4.2. Body Weight, Body Composition and Food Intake

Body weight and body composition were determined weekly by using an EchoMRI-100 analyzer (EchoMRI, Houston, TX, USA). Food intake was measured weekly by subtracting the amount of food that was left in the cage from the amount given at the previous weighing. 

### 4.3. Plasma Parameters

Before randomization and every three weeks thereafter, mice were fasted for four hours, and blood was drawn from the tail vein. Blood was collected in paraoxon-coated capillaries (Sigma, St. Louis, MO, USA) that were immediately placed on ice and centrifuged to obtain plasma. TG, total cholesterol (TC), and glucose were determined using commercially available enzymatic kits (Roche Diagnostics, Mannheim, Germany for TG and TC and Instruchemie, Delfzijl The Netherlands for glucose). FFA were measured using an NEFA C kit (Wako Diagnostics, Instruchemie, Delfzijl, The Netherlands).

### 4.4. Lipoprotein Profiles 

Plasma samples obtained after 12 weeks of intervention were pooled to determine cholesterol distribution over plasma lipoproteins by fast performance liquid chromatography (FPLC). Plasma was placed onto a Superose 6 column (ÄKTA System, Amersham Pharmacia Biotech, Piscataway, NJ, USA) and eluted at a constant flow of 50 µL/min with phosphate buffered saline pH 7.4. Individual fractions were measured for TC, as described above.

### 4.5. Indirect Calorimetry and Physical Activity

During the third week of treatment, mice (*n* = 8 per group) were placed in fully automatic metabolic cages (LabMaster System, TSE Systems, Bad Homburg, Germany) to assess energy expenditure and physical activity levels. After 20 h of acclimatization, oxygen uptake (V˙ O_2_) and carbon dioxide production (V˙ CO_2_) were measured for five consecutive days. Total energy expenditure (EE) was calculated from V˙ O_2_ and V˙ CO_2_ using the Weir equation [35]. Fat and glucose oxidation rates were calculated from V˙ O_2_ and V˙ CO_2_, as described previously [36]. Physical activity was measured using infrared sensor frames.

### 4.6. Feces Collection and Fecal FFA Concentration

After two and 10 weeks of intervention, feces was collected by placing the mice in cages with new bedding and removing feces 24 h later. Feces was then dried and weighed, approximately 30 mg was ground, and fecal FFA were determined after methyl esterification using the NEFA C kit (Wako Diagnostics), as described previously [37]. 

### 4.7. RNA Isolation and qRT-PCR Analysis

Total RNA was isolated using TriPure Isolation reagent (Roche obtained via Sigma, St. Louis, MO, USA ) following the manufacturer’s protocol. cDNA was made using Moloney Murine Leukemia Virus Reverse Transcriptase (Promega, Leiden, The Netherlands). Quantitative real-time PCR (qRT-PCR) was performed with SYBR green (Promega) on a CFX96 PCR Machine (Bio-Rad, Veenendaal, The Netherlands). mRNA expression levels were normalized to *β2-microglobulin* or glyceraldehyde-3-phosphate dehydrogenase (*Gapdh*) and hypoxanthine guanine phosphoribosyl transferase (*Hprt*) as reference genes. Primer sequences are listed in Table 1.

### 4.8. In Vivo Clearance of Radiolabeled Lipoprotein-Like Particles

Lipoprotein-like particles (80 nm) labeled with glycerol tri[^3^H]oleate (triolein, [^3^H]TO) were prepared and characterized as described previously [38]. Mice were fasted for 4 h and injected (*t* = 0) with 200 µL of [^3^H]TO-labeled lipoprotein-like particles (1.0 mg TG per mouse) via the tail vein. Blood samples were taken 2, 5, 10, and 15 min after injection to determine the plasma decay of [^3^H]TO (using calculations described in [39]). Next, mice were sacrificed by means of cervical dislocation and perfused with ice-cold heparin solution (0.1% *v*/*v* in PBS) via the heart. Organs were harvested, weighed, and the uptake of [^3^H]TO-derived radioactivity was quantified and expressed per gram or whole organ wet tissue weight.

### 4.9. Histology

Interscapular BAT, gonadal WAT (gWAT), subcutaneous WAT (sWAT), visceral WAT (vWAT), and liver were removed and directly placed in 4% paraformaldehyde, dehydrated, and embedded in paraffin. Hematoxylin and Eosin staining were performed using standard protocols. An overview of the studied fat depots can be found in Figure S5. Relative cell size and intracellular lipid content was quantified using ImageJ (version 1.49). UCP-1 staining on sWAT and BAT were performed as described before [39]. 

### 4.10. Quantification of Lipid Content in Liver 

Liver samples (approx. 50 mg) were homogenized in 10 µL of ice-cold methanol per mg of tissue. Lipids were extracted as described before [39]. Hepatic TG, TC, and PL concentrations were measured using commercial kits (as explained in Plasma parameters). Liver lipids were expressed per milligram of protein, which was determined using a Pierce BCA protein assay kit (Thermo Scientific, Rockford, IL, USA).

### 4.11. Protein Isolation and Western Blot

sWAT samples were lysed in buffer containing 50 mM of *N*-(2-Hydroxyethyl)piperazine-*N*’-2-ethanesulfonic Acid (HEPES, pH 7.6), 50 mM of NaF, 50 mM of KCl, 5 mM of NaPPi, 1 mM of ethylenediaminetetraacetic acid (EDTA), 1 mM of ethylene glycol tetraacetic acid (EGTA), 1 mM of dithiothreitol (DTT), 5 mM of β-glycerophosphate, 1 mM of sodium vanadate, 1% of Nonidet P-40, and protease inhibitors using cocktail tablets (Roche). Protein concentration was determined using the Pierce BCA Protein Assay kit (Thermo Scientific), and extracts were diluted in Laemli buffer. Next, 10 µg of sWAT protein was separated on a 10% polyacrylamide gel (#456-8035, Bio-Rad) by electrophoresis and turbo-blotted on nitrocellulose membranes (#170-4159, Trans-blot Turbo, Bio-Rad). Membranes were blocked for 1 h at room temperature in Tween-20 buffer with 5% non-fat dry milk. Next, membranes were incubated overnight with 1:1000 or 1:5000 (only for UCP-1 in BAT) diluted specific primary antibodies. Primary antibodies specific for AMPKα (#2532), p-AMPKα (Thr172, #2535), and α/β-Tubulin (#2148) were purchased from Cell Signalling Technology (Leiden, The Netherlands) and the antibody specific for UCP-1 (#U6382) was purchased from Sigma (St. Louis, MO, USA). A primary antibody specific for Gapdh was purchased from Santa Cruz Biotechnology (Heidelberg, Germany). Membranes were incubated with horseradish peroxidase-conjugated secondary antibodies for 1 h. Super Signal West Pico Chemiluminescent Substrate (Thermo Scientific, Rockford, IL, USA) was used to visualize bands on the ChemiDocTM Touch imaging system (Bio-Rad). The images were analyzed with Image lab software (version 5.2, Bio-Rad), and protein content was corrected for GAPDH (housekeeping protein).

### 4.12. Determination of mtDNA/nDNA Ratio

Total DNA was isolated from sWAT with QIAamp DNA Mini Kit (no. 51306; Qiagen, Hilden, Germany), following the provided manufacturer’s protocol. The PCR reaction was performed in a LightCycler 480 (Roche). The reaction mix contained 500 pg of DNA, 1.25 µM of forward and reverse primer, and 4 µL of 2× SYBR green master mix (Roche) in a final volume of 8 µL. Then, *16S* and *Cox2* were used as mitochondrial-encoded genes, and *Hk2* and *Ucp2* were used as nuclear-encoded genes. Primer sequences are listed in Table 2. Data were analyzed with LightCycler software release 1.5.0 (Roche). The mean PCR efficiency was calculated to assess gene expression levels using LinRegPCR program version 12.17 [40]. 

### 4.13. Statistical Analysis

All of the data are expressed as mean ± SEM. Statistical analysis was performed with an IBM SPSS Statistics 23 software package (SPSS, Chicago, IL, USA). The differences between control and quercetin were determined using an unpaired two-tailed *t*-test. Plasma decay in the clearance was analyzed using repeated ANOVA measurements with Sidak’s post hoc test. A *p*-value < 0.05 was considered statistically significant.

## Figures and Tables

**Table 1:** List of qRT-PCR primer sequences.

Gene	Forward Primer	Reverse Primer
*β2-microglobulin*	TGACCGGCTTGTATGCTATC	CAGTGTGAGCCAGGATATAG
*Angptl4*	GGAAAGAGGCTTCCCAAGAT	TCCCAGGACTGGTTGAAGTC
*Apob*	GCCCATTGTGGACAAGTTGATC	CCAGGACTTGGAGGTCTTGGA
*Acc2*	AGATGGCCGATCAGTACGTC	GGGGACCTAGGAAAGCAATC
*Acsl1*	TGCCAGAGCTGATTGACATTC	GGCATACCAGAAGGTGGTGAG
*Cd36*	GCAAAGAACAGCAGCAAAATC	CAGTGAAGGCTCAAAGATGG
*Cidea*	CTCGGCTGTCTCAATGTCAA	CCGCATAGACCAGGAACTGT
*Cyp3a11*	CTTTCCTTCACCCTGCATTCC	CTCATCCTGCAGTTTTTTCTGGAT
*Elovl3*	GGATGACGCCGTAGTCAGTA	GACAGAATGGACGCCAAAGT
*Gapdh*	GGGGCTGGCATTGCTCTCAA	TTGCTCAGTGTCCTTGCTGGGG
*Hprt*	TTGCTCGAGATGTCATGAAGGA	AGCAGGTCAGCAAAGAACTTATAG
*Lpl*	CCCTAAGGACCCCTGAAGAC	GGCCCGATACAACCAGTCTA
*Mttp*	CTCTTGGCAGTGCTTTTTCTCT	GAGCTTGTATAGCCGCTCATT
*Pgc1a*	TGCTAGCGGTTCTCACAGAG	AGTGCTAAGACCGCTGCATT
*Prdm16*	ACTTTGGATGGGAGCAGATG	CTCCAGGCTCGATGTCCTTA
*Pxr*	GAGCGGAGAAGACGGCAGCATC	CCCAGGTTCCCGTTTCCGTGTC
*Ucp1*	TCAGGATTGGCCTCTACGAC	TGCATTCTGACCTTCACGAC

**Table 2:** List of PCR primer sequences.

Gene	Forward Primer	Reverse Primer
*16S*	CCGCAAGGGAAAGATGAAAGAC	TCGTTTGGTTTCGGGGTTTC
*Cox2*	GTTGATAACCGAGTCGTTCTGC	CCTGGGATGGCATCAGTTTT
*Hk2*	TCTGGCTCTGAGATCCATCTTCA	CCGGCCTCTTAACCACATTCC
*Ucp2*	CTACAGATGTGGTAAAGGTCCGC	GCAATGGTCTTGTAGGCTTCG

## Supplementary Materials

Supplementary materials can be found at http://www.mdpi.com/1422-0067/19/6/1786/s1.

## Abbreviations

ACC2; *Acc2*	acetyl-CoA carboxylase 2
ACSL1; *Acsl1*	long-chain-fatty-acid-CoA ligase 1
AMPK	AMP-activated protein kinase
APOB; *Apob*	apolipoprotein B
ANGPTL4; *Angptl4*	angiopoietin-like 4
(i)BAT	(interscapular) brown adipose tissue
CD36; *Cd36*	fatty acid translocase
EE	energy expenditure
ELOVL3; *Elovl3*	fatty acid elongase 3
(F)FA	(free) fatty acids
HFD	high fat diet
LDLr	low density lipoprotein receptor
LPL; *Lpl*	lipoprotein lipase
LRP	low density lipoprotein receptor-related protein
MTP; *Mttp*	microsomal triglyceride transfer protein
PGC1α; *Pgc1α*	peroxisome proliferator-activated receptor γ coactivator 1α
PXR; *Pxr*	pregnane X receptor
RER	resting energy expenditure
SIRT1	sirtuin 1
TC	total cholesterol
TG	triglyceride
TO	triolein
UCP-1; *Ucp1*	uncoupling protein-1
VLDL	very-low density lipoprotein
(g,s,v)WAT	(gonadal, subcutaneous, visceral) white adipose tissue
