# Supplementary material for: Quercetin Lowers Plasma Triglycerides Accompanied by White Adipose Tissue Browning in Diet-Induced Obese Mice
Source: Int J Mol Sci. 2018 Jun 16;19(6):1786. doi: 10.3390/ijms19061786 (PMC6032193; doi:10.3390/ijms19061786)
Supplement: Supplementary file 1 [file ijms-19-01786-s001.pdf]

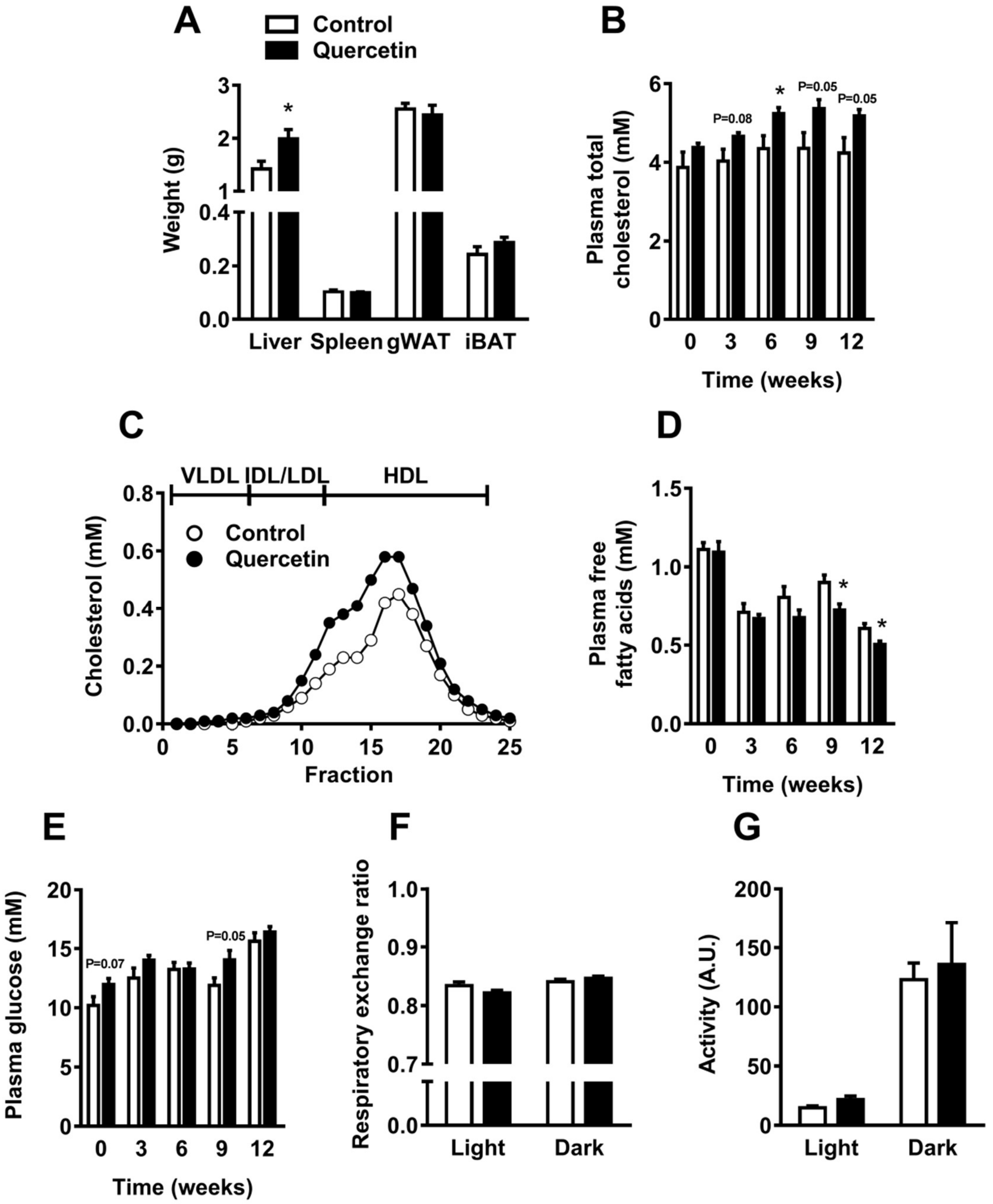

Figure S1. Quercetin increases liver weight, plasma, and total cholesterol, and decreases plasma free fatty acids. The organ weight of liver, spleen, iBAT, and gWAT (A) was determined at the end of the intervention. Plasma total cholesterol (B), free fatty acids (D), and glucose (E) were analyzed at the indicated time points. Cholesterol distribution over lipoproteins after separation from pooled plasma after 12 weeks of treatment was assessed by FPLC (C). In the third week of treatment, animals were placed in fully automatic metabolic cages, and respiratory exchange ratio (F) and activity (G) were determined. Data are represented as mean ± SEM (n = 8-10), * $p < 0.05$ versus control.

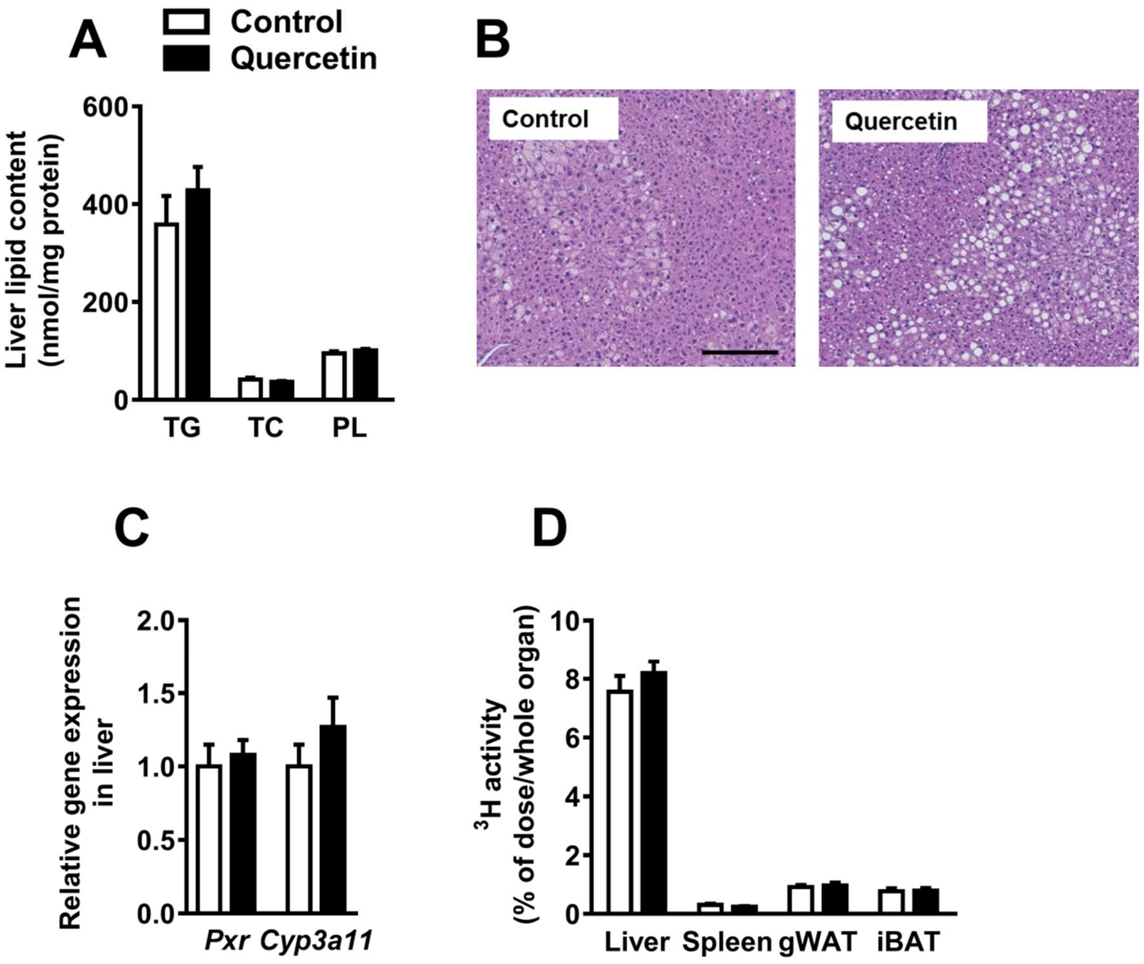

Figure S2. Quercetin does not affect liver lipids or whole liver TG-derived FA uptake. Liver was analyzed for triglyceride (TG), total cholesterol (TC), and phospholipid (PL) content (A). Representative pictures of liver sections stained for H&E from control and quercetin-treated animals (B, bar indicates 200μm). Gene expression in the liver was determined by qRT-PCR (C). After 12 weeks, mice were injected with glycerol tri[^{3}H]oleate-labeled lipoprotein-like particles, and uptake was determined by ^{3}H-activity corrected for whole organ weight (D). Data are represented as mean ± SEM (*n* = 8-10); expression of genes was corrected for the reference gene *β2-microglobulin*.

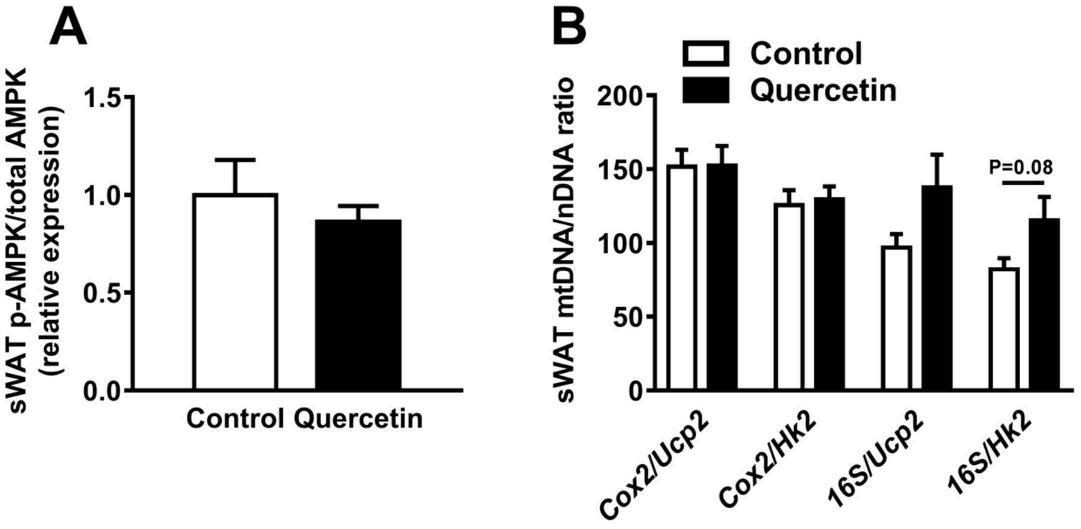

Figure S3. Quercetin does not affect the p-AMPK/total AMPK ratio, and tends to increase the 16s/Hk2 ratio in subcutaneous white adipose tissue. Protein levels were determined by Western blot in sWAT (A). Total DNA was isolated from sWAT and primers for mitochondrial and nuclear encoded genes were used to determine the mitochondrial DNA versus nuclear DNA ratio (mtDNA/nDNA, C) by PCR. Data are represented as mean ± SEM (n = 8-10); protein content was corrected for the reference protein glyceraldehyde-3-phosphate dehydrogenase (GAPDH).

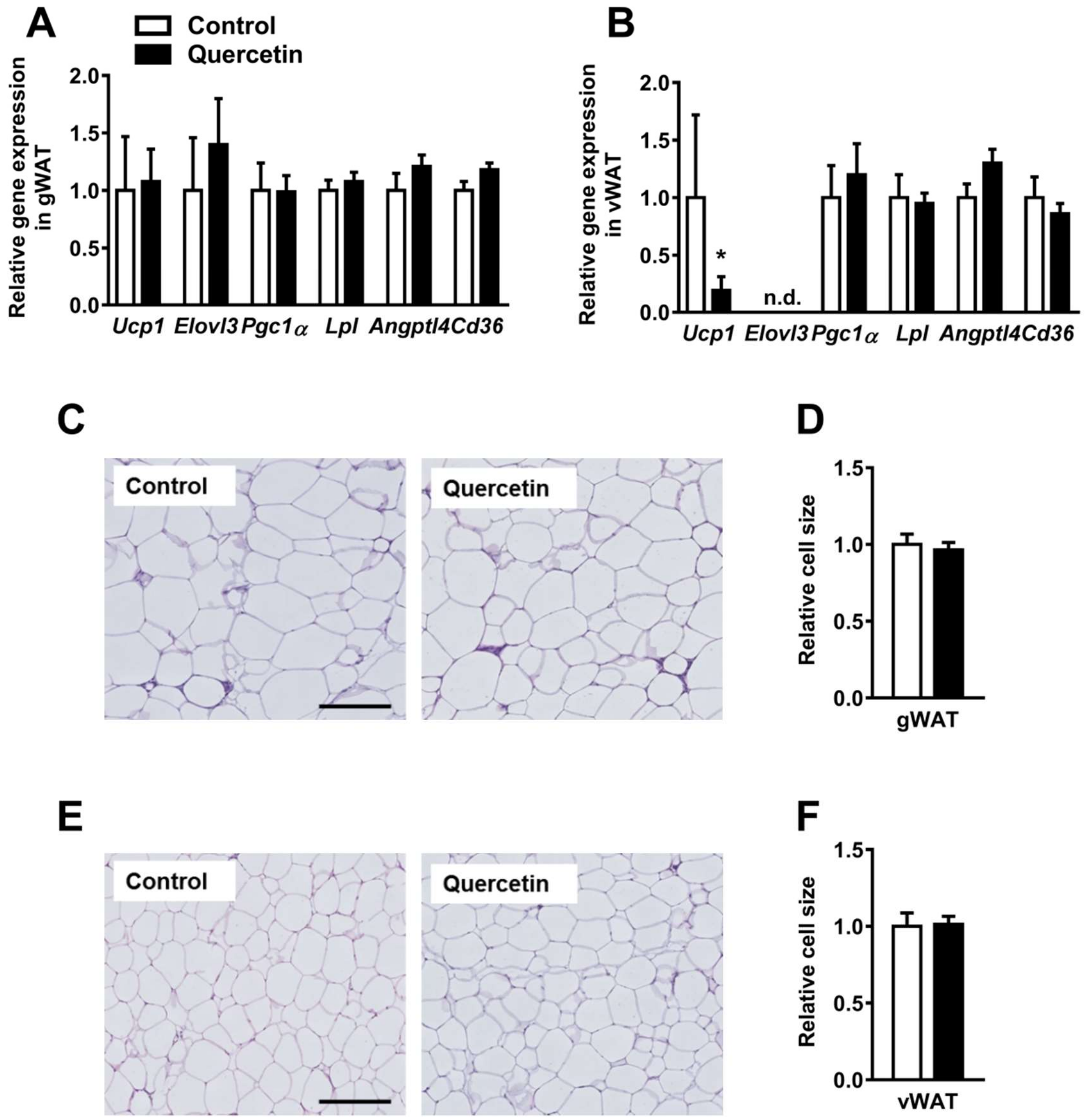

Figure S4. Quercetin does not affect browning markers in gonadal and visceral white adipose tissue. Gene expression was determined by qRT-PCR in gWAT (A) and vWAT (B). H&E staining was performed on sections of gWAT (C) and vWAT (E) from control and quercetin-treated animals, and representative pictures are shown. All pictures were used to determined relative cell size in gWAT (D) and vWAT (F) in ImageJ. Data are represented as mean ±SEM (n = 8-10); expression of genes was corrected for the reference gene *β2-microglobulin*. ND (not detected) indicates the average CT values were > 32. * $p < 0.05$ versus control. Bars (A and E) indicate 200 μm.

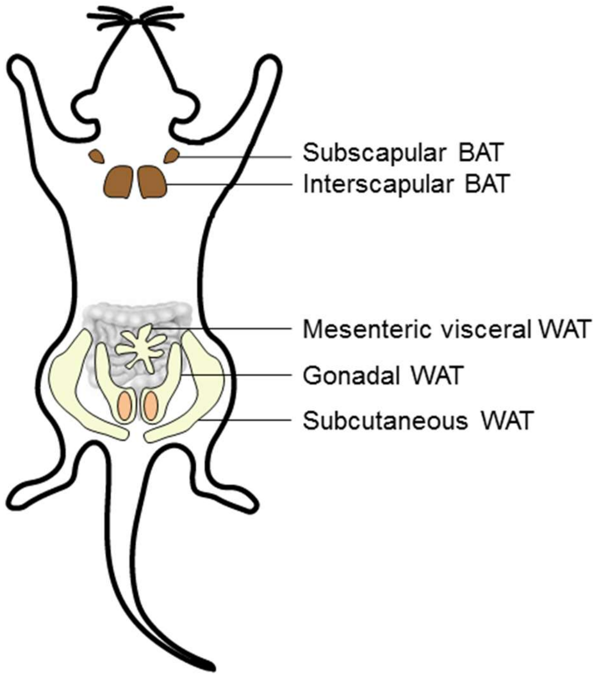

Figure S5. Schematic overview of studied fat depots. Subscapular BAT (sBAT), interscapular BAT (iBAT), mesenteric visceral WAT (vWAT), gonadal WAT (gWAT), and subcutaneous WAT (sWAT).
